# Evaluation of feeding effects of pelletized total mixed ration in Hu sheep: growth performance, bacterial community and rumen fermentation

**DOI:** 10.5713/ab.24.0852

**Published:** 2025-05-19

**Authors:** Chuankai Zhang, Xiaohui Kong, Peijun Hou, Tengyun Gao, Huaijun Zheng, Wenqing Li, Tong Fu, Liyang Zhang

**Affiliations:** 1 College of Animal Science and Technology, Henan International Joint Laboratory of Nutrition Regulation and Ecological Raising of Domestic Animals, Henan Agricultural University, Zhengzhou, China; 2 Gongzhuling Hefeng Ruminant Feed Co., Ltd, Gongzhuling, China; 3 College of Life Science, Henan Agricultural University, Zhengzhou, China

**Keywords:** Growth Performance, Pelleted Total Mixed Ration, Rumen Bacteria, Sheep

## Abstract

**Objective:**

The study aimed to compare the growth performance, rumen fermentation, and rumen bacterial community of fattening Hu sheep fed either total mixed ration (TMR) or pelleted total mixed ration (PTMR) and to assess the feeding efficiency.

**Methods:**

In a 58-day feeding experiment, forty-eight Hu sheep were randomly assigned to two groups (TMR and PTMR), with six pens per group and four sheep per pen. Body weight and feed intake were measured throughout the experiment to assess growth performance. On the final day of the experiment, rumen fluid was collected from sheep using a rumen fluid collector two hours post-feeding.

**Results:**

The sheep in the PTMR group exhibited significantly higher body weight (p<0.05) and average daily gain (p<0.01) compared to those in the TMR group, although the effect of PTMR on dry matter intake was not statistically significant (p>0.05). No statistically significant differences were observed in rumen fermentation parameters between the TMR and PTMR groups. However, analysis of rumen bacteria revealed that the Sob, Ace, Bootstrap, Shannon, and Chao indices were significantly lower in the PTMR group compared to the TMR group (p<0.05). *Bacteroidetes* and *Firmicutes* were the predominant bacteria in all groups, with the abundance of *Succinivibrionaceae_UCG-001* significantly lower in the PTMR group. The relative abundance of *Rikenellaceae_RC9_gut_group*, *NK4A214_group*, and *Streptococcus* was significantly higher in the TMR group than in the PTMR group (p<0.05). Bacterial function prediction showed downregulation of the energy production and conversion pathway in the PTMR group (p<0.05). Correlation analysis indicated that *norank_f_Bacteroidales_RF16* positively correlated with butyric acid (p<0.05), while *Anaerovibrio* negatively correlated with acetic acid, propionic acid, butyric acid, and total volatile fatty acids (p<0.05).

**Conclusion:**

In summary, the results demonstrate that PTMR significantly enhances average daily gain in Hu sheep while maintaining rumen fermentation parameters, although accompanied by modifications in rumen bacterial community structure.

## INTRODUCTION

The total mixed ration (TMR) feeding model is widely used in ruminant production due to its ability to provide animals with a balanced intake of required nutrients [1]. However, TMR diets cannot completely eliminate the selective feeding behavior of animals, and the high moisture content of TMR makes it inconvenient for storage, thus increasing labor costs [2,3]. To address these challenges, the pelleted total mixed ration (PTMR) process has emerged as a promising solution. Recent studies have demonstrated that PTMR not only reduces selective feeding behavior but also alters the nutrient structure during processing. Additionally, the extrusion process during pelletizing results in smaller particles, which reduces transportation and storage costs [4]. The smaller particle size also increases the bacterial attachment area, thereby enhancing substrate fermentation [5].

The nutritional value of pelleted diets is determined by its composition, which in turn affects the feeding efficiency of these diets [6]. By replacing corn with wheat intermediates in PTMR, the digestibility of neutral detergent fiber (NDF) can be improved [7]. The addition of TMR granules with different proportions of barley straw decreases the digestibility of dry matter (DM), but has no effect on the digestibility of acid detergent fiber (ADF), NDF and nitrogen balance [8]. When considering feed composition, the role of rumen microorganisms should not be overlooked. The fermentation products produced daily by rumen microorganisms are the direct sources of nutrients for the animal body. Compared to non-grain diets, diets with grains crushed to less than 8 mm can affect the adhesion of rumen microorganisms. Studies have shown that PTMR diets can improve the composition of rumen bacteria [9]. However, current research has not yet systematically assessed the impact of feeding a PTMR diet on rumen health, bacterial composition, and overall production performance in Hu sheep.

In this study, we hypothesized that the PTMR would affect the rumen fermentation associated with rumen digesta and growth performance in Hu sheep. Consequently, the objective of this study was to evaluate the impact of PTMR on growth performance, rumen fermentation, and the composition of rumen bacteria in Hu sheep. The ultimate goal was to provide fundamental insights that could inform the application of PTMR in the fattening of Hu sheep.

## MATERIALS AND METHODS

### Animals, diets, and experimental design

Forty-eight Hu sheep, 150 days of age with an average body weight (BW) of 31.40±0.55 kg (mean±standard deviation), were used for a 58-day feeding experiment. The sheep were assigned to two groups in a completely randomized design, with six pens (four sheep per pen) per group. Two diets with identical nutritional compositions were formulated: TMR and PTMR (Table 1). The TMR diet was prepared by chopping dried peanut vines into 3–4 cm pieces and thoroughly mixing them with concentrate feed at a 1:1 ratio (wt/wt). The PTMR diet, containing the same 50:50 ratio of chopped peanut vines to concentrate feed, was processed into pellets using a pelleting machine (9KLP-300; Henan Hengfu Machinery Equipment, Xinxiang, Henan, China) with a pellet diameter of 6 mm. A single batch of feed was prepared for the entire experimental period.

Hu sheep were fed twice daily (at 07:00 and 17:00) on an *ad libitum* basis during the experimental period. Daily feed residues were weighed, and the feed supply was adjusted to maintain a 5% daily residue. Each pen was equipped with a separate drinking bowl to provide unlimited access to water.

### Laboratory analysis for feed and ort samples

Each sample was dried in a laboratory oven at 60°C for 24 h to a constant weight, then ground using a mill and sieved through a 1 mm mesh for further analysis. DM, crude ash (Ash), calcium (Ca), and phosphorus (P) contents were determined according to the AOAC method [10]. Crude protein (CP) content was determined by Kjeldahl analysis using a Kjeltec 2300 analyzer (FOSS Analytical AB, Hoganas, Sweden). NDF and ADF contents were analyzed according to Van Soest et al [11] using an Ankom Fiber Analyzer (Ankom Technology, Fairport, NY, USA).

### Intake and growth performance

The daily TMR from each pen was weighed and sampled every day. Before the morning feeding on days 0, 28, and 56 of the feeding experiment, all sheep were weighed. Average daily gain (ADG), dry matter intake (DMI), and feed conversion ratio (F/G) were calculated using the aforementioned measurements.

### Collection of rumen samples

Ruminal fluid samples were collected from each sheep two hours after morning feeding on day 56 of the experiment as previously described [12]. Samples were aliquoted into cryopreservation tubes, immediately frozen in liquid nitrogen, and transported to the laboratory for storage at −80°C.

### Ruminal fermentation analysis

Ruminal fluid pH was measured immediately after sample collection using a Russell CD700 pH meter. Two samples from each pen were randomly selected for ruminal volatile fatty acids (VFA) analysis. The samples were centrifuged at 10,000×g for 15 minutes at 4°C to pellet particulate matter. The supernatant was then filtered through a 0.45-μm membrane for clarification. Samples were treated with 25% (w/v) metaphosphoric acid to stabilize VFA at a final concentration of 2% (w/v). Acid-treated samples were centrifuged again under the same conditions (10,000×g, 10 minutes, 4°C) to remove protein precipitates. Samples were stored at −20°C and thawed for 30-fold dilution with deionized water immediately before analysis. VFA analysis was performed using an ICS-3000 ion chromatography system with an IonPac AS11-HC column (4×250 mm). Chromatographic separation used a 0.5 mL/min flow rate of 30 mM KOH eluent with the column temperature held at 35°C. Calibration curves were prepared from mixed standard solutions containing acetic, propionic, and butyric acids at 50, 100, 150, and 200 mg/L. A 20 μL aliquot of each diluted sample was injected and quantification was performed using external standards. Ruminal NH_3_-N concentration was determined using UV spectrophotometry.

### Ruminal bacterial community analysis

Seven samples per group were randomly selected from the aforementioned 12 samples per group for 16S rRNA gene sequencing. Rumen fluid samples were processed for DNA extraction and subsequent microbial community analysis. Total bacterial DNA was extracted using a QIAamp DNA Stool Mini Kit (Qiagen, Hilden, Germany) according to the manufacturer’s protocol. The 16S rRNA gene was then amplified with the universal primer pair (5′-ACTCC TACGGGAG GCAGCA-3′ and 5′-GGACTACHVGGGTWTCTAAT-3′) spanning the V3-V4 hypervariable region [13]. The integrity of the PCR product was verified and it was purified, followed by measurement of its concentration. The PCR products were then pooled in equal proportions based on their DNA concentrations. Sequencing of the 16S rRNA gene amplicons was performed using the Illumina HiSeq 2500 system (Illumina, San Diego, CA, USA) [14].

### Bioinformatics analysis

Raw sequencing data were processed through the following steps: 1) denoising, 2) paired-end read merging with FLASH v1.2.11, 3) quality filtering using Trimmomatic v0.33, and 4) chimera removal with UCHIME v8.1. Filtered reads were clustered into operational taxonomic units (OTUs) at 97% sequence similarity with USEARCH v10.0, against the Greengenes database (release 13_8). Taxonomic annotation of representative OTU sequences was performed using the RDP Classifier with a confidence threshold of 0.8 [15]. Abundance was normalized based on the least sequenced sample. Alpha diversity indices and rarefaction curves were generated in MOTHUR v1.3.0. Beta diversity was visualized through principal coordinates analysis (PCoA) based on weighted UniFrac distance matrices. Functional potential was predicted from 16S rRNA gene data using PICRUSt2, with KEGG pathways and COG categories annotated for metabolic and functional profiling respectively.

### Statistical analysis

Statistical analyses were performed using SAS v9.2 software (SAS Institute, Cary, NC, USA). Daily intake measurements were aggregated into monthly means. BW, DMI and ADG data were analyzed using a repeated-measures mixed model with a randomized complete design. The model incorporated fixed effects of treatment, time (days on-feed), and treatment× time interaction, with pen as a random effect and sheep within a pen. Independent-samples t-tests were performed in IBM SPSS Statistics 26 (IBM, Armonk, NY, USA) to compare ruminal VFA concentrations and alpha diversity indices (ACE, Chao1, Shannon, Simpson), and significance was declared when p<0.05. Bacterial community differences were assessed by linear discriminant analysis (LDA) effect size (LEfSe), with a significance threshold of LDA score>3.0. Spearman’s rank correlation coefficient was used for correlation testing.

## RESULTS

### Intake and growth performance

The effects of feeding PTMR diets on sheep intake and growth performance were presented in Table 2. The PTMR group had significantly higher BW (p<0.05) and showed a significant effect on ADG (p<0.05). However, the PTMR group had no significant effect on DMI (p = 0.115) or F/G (p = 0.866). Significant time-treatment interaction effects were observed for DMI (p<0.05), with the PTMR group exhibiting increased intake over time. Longitudinal analysis revealed significant temporal variations in BW, ADG, DMI, and F/G between PTMR and TMR groups throughout the experiment (p<0.05).

### Ruminal fermentation analysis

No significant differences were observed in ruminal fermentation parameters between TMR and PTMR groups, including pH (p = 0.429), acetate (p = 0.270), propionate (p = 0.321), butyrate (p = 0.302), acetate/propionate (p = 0.985), total VFA concentrations (p = 0.444), or NH_3_-N (p = 0.210) (Table 3).

### Ruminal bacterial community composition

**Figure 1:**
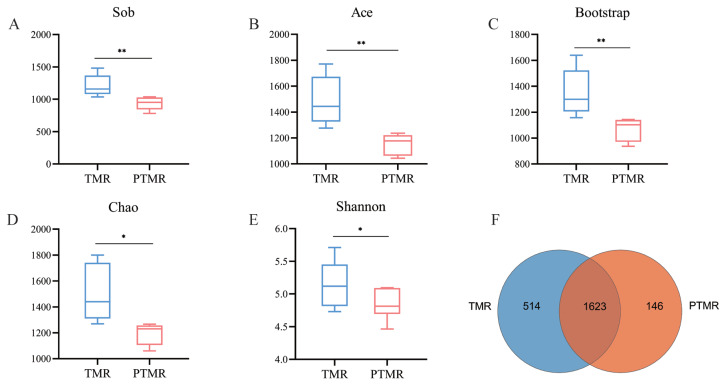
Analysis of rumen microbial diversity of sheep in the TMR and PTMR groups by 16S rRNA gene sequencing. Alpha diversity analysis (A–E), (F) OTU-Venn diagram analysis of TMR and PTMR. TMR, total mixed ration; PTMR, pelleted total mixed ration; OTU, operational taxonomic unit. * p<0.05, ** p<0.01.

16S rRNA gene sequencing yielded 47,800±1,688 high-quality reads per sample (mean±standard error of the mean). The TMR group exhibited significantly higher alpha diversity indices (Sob, Ace, Bootstrap, Shannon, and Chao) than the PTMR group (p<0.01), including Shannon, Chao1, ACE, and Simpson indices. A Venn diagram showed 1,623 shared OTUs between groups, with 514 OTUs unique to TMR and 146 OTUs specific to PTMR (Figure 1).

**Figure 2:**
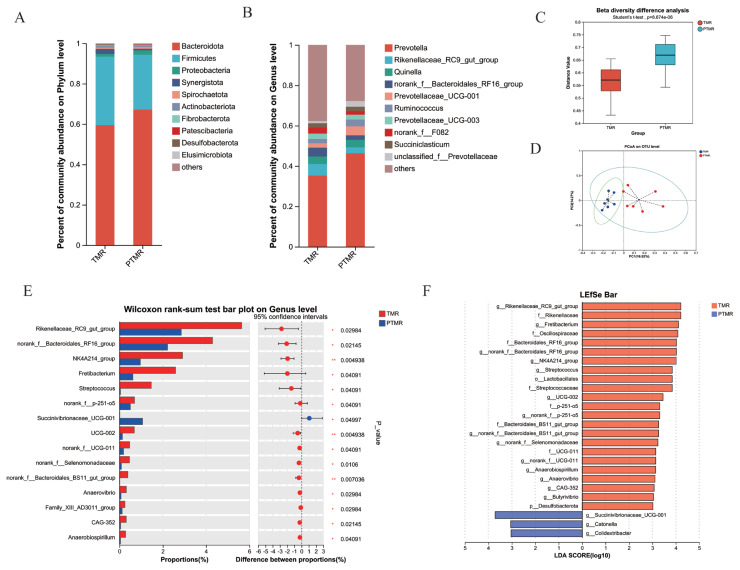
(A) Relative abundance of phylum level species. (B) Relative abundance of genus level species. (C) Bata diversity difference analysis of TMR and PTMR. (D) OTU-PCoA analysis of TMR and PTMR. (E) Wilcoxon rank-sum test bar plot on genus level of TMR and PTMR. (F) Linear discriminant analysis (LDA) value distribution histogram. LDA value>3, the length of the bar chart represents the influence of different species. TMR, total mixed ration; PTMR, pelleted total mixed ration; OTU, operational taxonomic unit; PCoA, principal coordinates analysis.

The relative abundance of *Bacteroidetes* in both TMR and PTMR was greater than 59%, while the relative abundance of *Firmicutes* was greater than 27%, accounting for more than 70% of the total abundance (Figure 2A). In addition, *Desulfobacterota* had a significantly greater relative abundance in TMR than in PTMR (p<0.05). The relative abundance of *Desulfobacterota* in TMR accounted for 3.12%, while in PTMR it was 1%. At the genus level, *Prevotella* was the dominant genus in the rumen of both the TMR and PTMR groups (Figure 2B). A total of 45 different species were identified between the two groups, with significant differences (p<0.05). Taking the top fifteen different species as an example, the relative abundance of *Rikenellaceae_RC9_gut_group*, *norank_f__Bacteroidales_RF16_group*, *NK4A214_group*, *Fretibacterium*, *Streptococcus*, *norank_f__p-251-o5*, *UCG-002*, *norank_f__UCG-011*, *norank_f__Selenomonadaceae*, *norank_f__Bacteroidales_BS11_gut_group*, *Anaerovibrio*, *Family_XIII_AD3011_group*, *CAG-352*, and *Anaerobiospirillum* was significantly higher in TMR than in PTMR (p<0.05) (Figure 2D). Additionally, the relative abundance of *Succinivibrionaceae_UCG-001* was significantly higher in PTMR than in TMR (p<0.05). LEfSe analysis of samples from the two groups (Figure 2F) revealed that there were 26 differential biomarkers (LDA score>3) for TMR and PTMR.

### Prediction of rumen bacterial function

**Figure 3:**
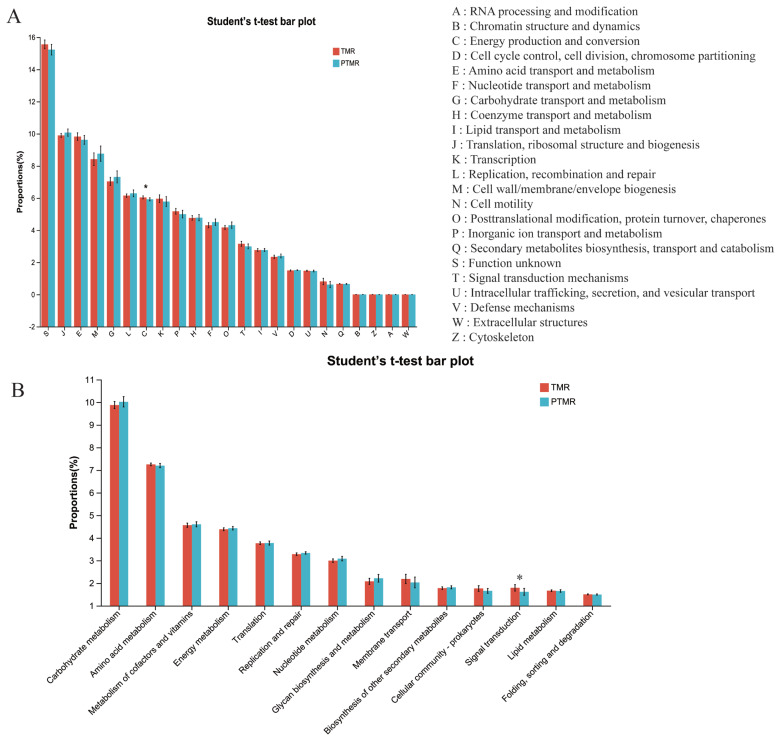
Gene Ontology (GO) and Kyoto Encyclopedia of Genes and Genomes (KEGG) enrichment analysis. (A) Gene Ontology (GO) enrichment analysis of TMR and PTMR. The figure shows 23 GO analysis enriched secondary classifications. (B) Kyoto Encyclopedia of Genes and Genomes (KEGG) enrichment analysis of TMR and PTMR. The figure shows the top 14 KEGG enriched secondary classifications with the highest abundance. TMR, total mixed ration; PTMR, pelleted total mixed ration. * p<0.05.

Functional profiles predicted from 16S rRNA gene data revealed 39 KEGG pathways and 23 COG categories. Among the 23 COG categories, “Energy production and conversion” was the sole functional class showing significant intergroup differences between TMR and PTMR (Figure 3A). Similarly, of the 39 KEGG pathways, “Signal transduction” was the only one that exhibited significant differences between TMR and PTMR (p<0.05). Metabolic pathways predominated in functional classifications, with carbohydrate metabolism being the most predominant subcategory, followed sequentially by amino acid metabolism and energy metabolism. None of these metabolic subcategories showed significant intergroup differences (Figure 3B).

### Interactions between rumen bacterial, average daily gain, dry matter intake and volatile fatty acids

**Figure 4:**
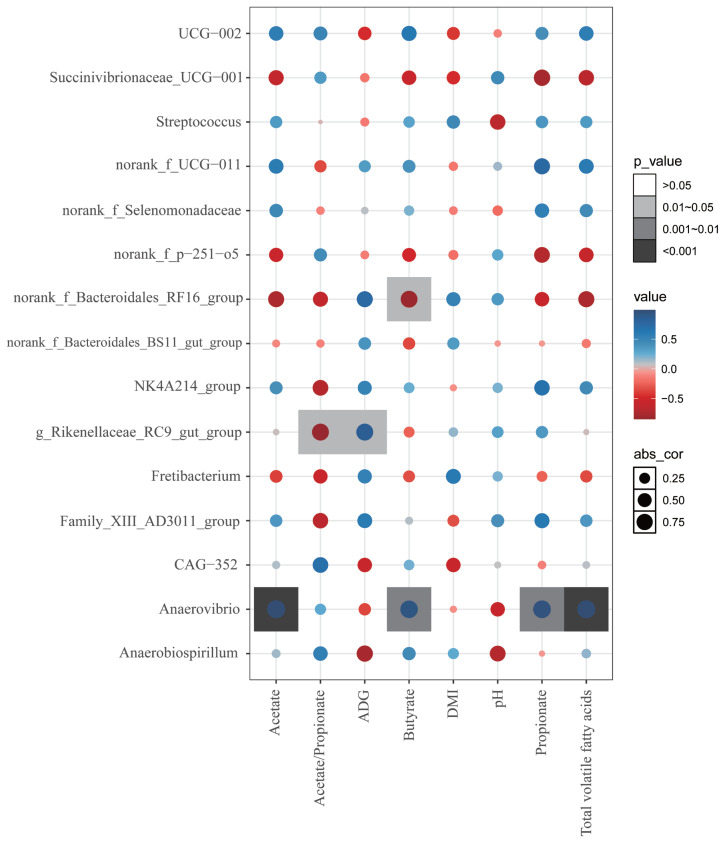
Correlation analysis between differential bacterial genera, average daily weight gain (ADG), dry matter intake (DMI), pH and volatile fatty acids (VFA).

As shown in Figure 4, the correlation heatmap was constructed based on rumen bacteria, specifically the top 15 genus level bacteria that exhibited significant differences according to p-values. The result revealed that VFA were significantly correlated with three genus level bacteria, of which two were positively correlated and one was negatively correlated (p<0.05). Furthermore, *Anaerovibrio* showed significant negative correlations with acetic acid, butyrate, propionate and total VFA (p<0.05). Moreover, *Rikenellaceae_RC9_gut_group* showed significant positive correlations with Acetate/Propionate and significant negative correlations with ADG (p<0.05). In addition, butyrate was positively correlated with *norank_f__Bacteroidales_RF16_group* of bacteria.

## DISCUSSION

### Pelleting on the growth performance

Dietary physical form significantly influences ruminant growth performance. Our results demonstrated that Hu sheep receiving PTMR exhibited higher ADG compared to those fed conventional TMR. This observation aligns with previous reports that pelleted diets enhanced growth performance in small ruminants [16,17]. Notably, while the DMI of the PTMR group was not significantly different from that of the TMR group overall, a significant time-treatment interaction was observed. The difference may be attributed to rumen adaptation to PTMR diets, as Zhong et al reported higher DMI in PTMR groups compared with our findings [18]. The PTMR group achieved higher ADG, suggesting that pelleting improves growth performance through mechanisms potentially related to enhanced rumen escape of nutrients. High density and sinking velocity may promote higher rumen escape rates in PTMR-fed sheep [19]. The enhanced ADG observed in pelleted diet studies may be linked to shifting nutrient digestion patterns toward the hindgut, a mechanism hypothesized to improve metabolic efficiency. It is noteworthy that PTMR-fed sheep showed only a non-significant DMI trend alongside a significant ADG increase, suggesting that pelleting subtly enhances growth performance through mechanisms beyond the current study’s scope. Further research measuring digestibility and rumen dynamics is needed to clarify these mechanisms.

### Pelleting on rumen fermentation

The pH and VFA concentrations in rumen fluid serve as critical indicators for evaluating ruminal fermentation, reflecting both the stability of the rumen environment and fermentation efficiency. In the present study, the PTMR exhibited no significant effects on rumen pH or VFA concentrations between the experimental groups, which aligns with the findings reported by Karimizadeh et al [20]. Similarly, Li et al [21] observed unchanged rumen VFA concentrations but a decreased rumen pH in lambs fed PTMR for 3 hours, attributing this pH reduction to the accelerated feeding rate associated with PTMR. These results suggest that the degradation and fermentation processes of PTMR in the rumen reach a stable state compared to conventional TMR, although granulation may simultaneously increase the rumen passage rate [22]. In this study, the rapid passage rate of PTMR might have shifted the primary site of fermentation from the rumen to the hindgut, where undigested substrates could undergo further microbial metabolism. This mechanism could explain the absence of significant changes in rumen VFA concentrations despite the observed improvement in average ADG. In contrast, Zhang et al [23], reported significant differences in acetic, propionic, and butyric acid concentrations even in post-slaughter measurements after 12 hours of PTMR feeding, hypothesizing that pelletization enhances feed intake rate and stimulates microbial fermentation activity. These divergent findings highlight the need for more systematic and dynamic measurements of rumen pH and VFA profiles in future studies to comprehensively elucidate the effects of granular TMR on rumen fermentation kinetics.

### Pelleting on rumen bacterial community

The regulation of metabolic functions of the body through the interaction between the host and bacteria has been extensively studied. Dietary habits are among the crucial factors that contribute to bacterial diversity and community composition, ultimately influencing body health [24]. Nutritional intervention can influence estrogen levels, modulate the gut microbiota, and facilitate increased dietary microbiota diversity [25]. The present study sequenced the rumen bacteria in Hu sheep and found that the three most abundant bacterial phyla were *Bacteroidetes*, *Firmicutes*, a*nd Proteobacteria*, which is consistent with the findings reported by Zhang et al [23]. In ruminants, Firmicutes and Bacteroidetes play a crucial role in fiber degradation and the digestion of complex carbohydrates [26]. The most abundant bacteria at the genus level included *Prevotella*, *Rikenellaceae RC9 gut group*, *Quinella*, *norank f Bacteroidales RF16 group*, *Prevotellaceae UCG 001*, *Ruminococcus*, *Prevotellaceae UCG 003* and Succiniclasticum group. These bacterial genera remained unaffected by the physical forms investigated in this study. The stability of these abundant bacterial genera may indicate the presence of a core microbiome [27]. In the present study, we observed that the relative abundance of the *Rikenellaceae RC9 gut group* at the genus level was significantly higher in TMR compared to PTMR, which aligns with the findings reported by Bo Trabi et al [28]. Conte et al found that the *Rikenellaceae RC9 gut group* is associated with lipid metabolism in the bovine rumen [29]. Moreover, the relative abundance of *norank f Bacteroidales RF16 group*, *NK4A214 group, Fretibacterium*, *Streptococcus, UCG-002*, and other bacteria was significantly higher in TMR compared to the PTMR group at the genus level. This may be attributed to the longer retention time of TMR in the rumen, allowing for more extensive fermentation by fiber-degrading bacteria such as *Rikenellaceae RC9*, as well as the increased activity of other bacterial genera (e.g., *norank_f_Bacteroidales_RF16_group, NK4A214_group, Fretibacterium, Streptococcus*, and *UCG-002*) involved in nutrient metabolism processes, including carbohydrate and fatty acid metabolism [30,31]. In contrast, *Succinivibrionaceae UCG-001* was the only bacterial genus in the PTMR group that exhibited a significantly higher relative abundance compared to the TMR group. An increase in the abundance of *Succinivibrionaceae* may significantly enhance the fermentation environment in the rumen, thus improving the digestibility and utilization of feed [32]. This correlation may be attributed to alterations in the physical properties of starch in TMR resulting from granulation treatment, which could promote the growth of starch-degrading bacteria such as *Succinivibrionaceae*. Although the rapid passage rate of PTMR in the rumen generally reduces the retention time for bacterial fermentation, *Succinivibrionaceae*, as a starch-degrading bacterium, may have a competitive advantage in utilizing the rapidly available starch substrates in PTMR, leading to its increased relative abundance [33]. Additionally, Zhang et al [23] found that PTMR feeding decreased cecal microbial abundance (e.g., *Lachnospiraceae*), indicating its modulatory role in hindgut metabolic processes. These findings suggest that granulation not only reduces the retention time of feed in the rumen but also selectively enriches bacterial taxa that are adapted to the altered dietary conditions, thereby influencing both rumen fermentation and nutrient utilization. Further in-depth research is warranted to elucidate the precise mechanisms underlying these observations.

Previous studies have reported changes in the response of microbial community composition and function to dietary alterations [34]. Consequently, we speculated that the bacterial abundance and functional profiles might respond to PTMR treatment. The prediction of bacterial gene functions further revealed distinct differences between Hu sheep fed TMR and those fed PTMR. Within the COG gene family, a significant decrease in energy production and conversion was observed in PTMR-fed animals. Relevant studies have reported a strong correlation between energy utilization and production performance in bacteria when subjected to changes in dietary nutrient composition [35]. In the present study, no significant alteration in rumen VFA concentration was observed in the PTMR group, whereas a notable difference was detected in the prediction of rumen bacterial functions. We hypothesize that this discrepancy may arise from alterations in energy supply due to the short-term retention of feed in the rumen following PTMR treatment. The rapid passage rate of PTMR likely reduced the extent of rumen fermentation, shifting the site of nutrient digestion and energy production to the hindgut. Such alterations may, to some extent, be attributed to changes in dietary energy density and substrate availability following pelletization.

Furthermore, we performed a prediction of bacterial functions based on the KEGG database, and the results revealed that signaling pathways were significantly downregulated in the PTMR group. This finding suggests that pelletization may influence the regulatory mechanisms of rumen bacteria, potentially affecting their ability to sense and respond to dietary changes. Despite the scarcity of relevant literature on the signaling role of rumen bacteria, the crosstalk among rumen bacteria and between rumen bacteria and the host constitutes one of the crucial regulatory mechanisms in ruminants [36].

Correlation analysis between rumen bacteria and the metabolites VFA, ADG, and DMI revealed significant associations among these variables. Prior studies have demonstrated a significant and positive correlation between *norank_f_Bacteroidales_RF16 group* and both propionate and acetate [37]. Another correlation analysis indicated that *Anaerovibrio* exhibited a significant negative correlation with acetic acid, propionic acid, butyric acid, as well as total VFA. This finding contrasts with previous studies, which have reported a positive correlation between *Anaerovibrio* and VFA production due to its role in lipid hydrolysis [38]. However, Chen et al also reported contrasting findings, showing that *Anaerovibrio* was positively correlated with the VFA [39]. In the present study, we hypothesize that under PTMR feeding conditions, the rapid passage rate of granular feed may have limited the extent of lipolysis by *Anaerovibrio*, thereby reducing its contribution to VFA production. Additionally, the altered physical properties of lipids in pelletized feed could further restrict substrate accessibility for *Anaerovibrio*, leading to the observed negative correlation. These results highlight the complex interplay between dietary form, bacterial activity, and metabolic outcomes in the rumen.

## CONCLUSION

This study demonstrated that PTMR significantly improves the growth performance of Hu sheep by increasing ADG and BW, while DMI remained statistically unchanged between treatments. PTMR altered the rumen bacterial community structure, notably increasing *Succinivibrionaceae UCG-001* and downregulating the energy production and conversion pathway. Additionally, *norank_f_Bacteroidales_RF16* was positively correlated with butyric acid, while *Anaerovibrio* showed a negative correlation with total VFA. These results suggest that PTMR enhances nutrient utilization through alterations in the rumen bacterial community, although further research is needed to clarify the underlying mechanisms.

## Figures and Tables

**Table 1:** Ingredients and chemical compositions of experimental diets

Item (% of DM)	Treatments	

	TMR	PTMR
Ingredients		
Corn	25.00	25.00
Wheat bran	6.50	6.50
Soybean meal	15.00	15.00
Premix^1)^	3.00	3.00
NaHCO_3_	0.50	0.50
Peanut vine	50.00	50.00
Total	100.00	100.00
Nutrient levels		
CP	15.54	15.44
NDF	31.11	27.01
ADF	20.35	17.24
Ash	10.00	10.62
Ca	1.18	1.27
P	0.19	0.21
DE (MJ/kg)^2)^	11.24	11.25

1) Premix provides the following per kg for diets: 1.0×10^5^ IU Vitamin A, 1.0×10^4^ IU Vitamin D_3_, 125 IU Vitamin E, 250 mg nicotinic acid, 75 mg pantothenic acid, 5 mg biotin, 50 mg Cu (as copper sulfate), Fe (as ferrous sulfate) 600 mg, Mn (as manganese sulfate) 500 mg, Zn (as zinc sulfate) 500 mg, I (as potassium iodide) 8.75 mg, Se (as sodium sulfate) 3.75 mg, Co (as cobalt sulfate) 3.75 mg.

2) Calculated value.

TMR, total mixed ration; PTMR, pelleted total mixed ration; DM, dry matter; CP, crude protein; NDF, neutral detergent fiber; ADF, acid detergent fiber.

**Table 2:** Feed intake and growth performance of Hu sheep fed PTMR and TMR diets

Item	Treatment		SEM	p-value		
	
	TMR	PTMR		Treatment	Time	Treatment×Time
BW (kg)	43.24	45.29	0.764	0.041	<0.001	0.331
ADG (kg day^−1^)	0.214	0.250	0.008	0.002	0.014	0.618
DMI (kg day^−1^)	1.62	1.75	0.049	0.115	<0.001	0.034
Ratio of feed to gain (F/G)	6.72	6.86	0.590	0.866	0.012	0.667

PTMR, pelleted total mixed ration; TMR, total mixed ration; SEM, standard error of the mean; BW, body weight; ADG, average daily weight gain; DMI, dry matter intake.

**Table 3:** Ruminal pH, volatile fatty acids, and ammonia nitrogen concentrations of Hu sheep fed with PTMR and TMR

Item	Treatment		SEM	p-value

	TMR	PTMR		
pH	6.46	6.56	0.121	0.429
Acetate (mmol L^−1^)	82.37	74.65	6.610	0.270
Propionate (mmol L^−1^)	35.15	31.49	3.505	0.321
Butyrate (mmol L^−1^)	16.19	18.57	2.194	0.302
Acetate/propionate	2.34	2.37	0.084	0.985
Total volatile fatty acids (mmol L^−1^)	133.71	124.71	8.992	0.444
Ammonia nitrogen (mg dL^−1^)	21.17	16.06	3.840	0.210

PTMR, pelleted total mixed ration; TMR, total mixed ration; SEM, standard error of the mean.
